# Two Childhood Pheochromocytoma Cases due to von Hippel-Lindau Disease, One Associated with Pancreatic Neuroendocrine Tumor: A Very Rare Manifestation

**DOI:** 10.4274/jcrpe.5078

**Published:** 2018-05-18

**Authors:** Aydilek Dağdeviren Çakır, Hande Turan, Ayça Aykut, Asude Durmaz, Oya Ercan, Olcay Evliyaoğlu

**Affiliations:** 1 İstanbul University Cerrahpasa Faculty of Medicine, Department of Pediatric Endocrinology, İstanbul, Turkey; 2 Ege University Faculty of Medicine, Department of Medical Genetics, İzmir, Turkey

**Keywords:** von Hippel-Lindau syndrome, pheochromocytoma, pancreatic neuroendocrine tumor, hemangioblastoma

## Abstract

von Hippel-Lindau (VHL) disease is an autosomal dominantly inherited disorder, characterized by hemangioblastomas of the retina and central nervous system (CNS); renal cysts; clear cell carcinoma; pheochromocytoma (PCC); endolymphatic sac tumors; cystadenomas of the epididymis in males; broad ligament of uterus in females; pancreatic cysts; cystadenomas; and neuroendocrine tumors. We report two cases of VHL disease that presented with PCC as the first manifestation. Further clinical developments during follow-up, hemangioblastoma of CNS in one case and a pancreatic neuroendocrine tumor (PNET) in the second case led to the diagnosis of VHL disease. Genetic analyses of the two cases revealed p.Arg161Gln (c.482G>A) and p.Leu129Pro (c.386T>G) heterozygous missense mutations in the VHL gene, respectively. In children, PCC may be the only and/or initial manifestation of VHL with delayed manifestations of the syndrome in other organs. PNET is a very rare manifestation of VHL disease. To the best of our knowledge, this is only the second reported case presenting with a combination of a PNET and bilateral PCC as components of childhood VHL disease. Pediatric patients diagnosed with PCC should be investigated for genetic causes and especially for VHL.

## What is already known on this topic?

In childhood, pheochromocytomas (PCC) are mostly due to genetic causes, of which von Hippel-Lindau (VHL) disease is the most frequent disorder. VHL may be the only and/or initial manifestation of the disease, with delayed manifestations of the syndrome in other organs.

## What this study adds?

We report two cases of von Hippel-Linda (VHL) disease who presented with pheochromocytomas (PCC). In the second case, pancreatic neuroendocrine tumor (PNET), a very rare manifestation of VHL disease, developed during follow-up. To the best of our knowledge, this is only the second case in the literature presenting with a combination of PNET and PCC in childhood.

## Introduction

von Hippel-Lindau (VHL) disease is an autosomal dominantly inherited disorder caused by a germline mutation in the VHL tumor supressor gene. VHL is characterized by hemangioblastomas of the retina and central nervous system (CNS); renal cysts; clear cell carcinoma; pheochromocytomas (PCC); endolymphatic sac tumors; cystadenomas of the epididymis in males and broad ligament of uterus in females; pancreatic cysts, cystadenomas and neuroendocrine tumors (1,2). Incidence of VHL disease is estimated at 2-3 cases per 100 000 population (3). If a family history of VHL disease is present, a diagnosis of VHL disease can be made by finding only a single VHL-associated tumor. On the other hand, approximately 20% of VHL cases are sporadic and in these cases the presence of two VHL tumors is necessary to diagnose the disease, in the absence of a positive family history (4).

PCCs are uncommon neuroendocrine tumors that arise from chromaffin cells of the adrenal medulla and produce excessive amounts of catecholamines, which are responsible for hypertensive surges, palpitations, headache, and diaphoresis (5). PCCs are rare in childhood but represent a curable cause of hypertension and must be considered in the differential diagnosis of hypertension. Compared with adults, children with PCCs have a higher incidence of bilateral adrenal tumors, extra-adrenal tumors and multiple tumors (6). Although most PCC cases are sporadic, more than 25% are associated with an inherited mutation and this ratio can be as high as 55%, if diagnosed before 18 years of age (7). In childhood, PCCs are mostly due to genetic causes, in which VHL disease is the most frequent disorder (8). PCCs in VHL disease tend to be seen at younger ages, are often multiple and may be extra-adrenal (9,10). 

Here we report two cases of VHL disease, who presented with PCC as the first manifestation. Further clinical developments during follow-up, hemangioblastoma of CNS in the first case and pancreatic neuroendocrine tumor (PNET) in the second case, led to the diagnosis of VHL disease. 

## Case Reports

### Case 1

This patient was a twelve year old boy, admitted with complaints of weight loss, hot flushes, palpitation and diaphoresis, for the past one month. He was the first child of nonconsanguineus parents. His birth history was unremarkable. His family history was not significant for tumor occurrence. On physical examination, he weighed 43kg [-0,28 standard deviation (SD)]. Height was 150 cm (-0,48 SD). His blood pressure was 140/100 mmHg (95p 123/81 mmHg), heart rate was 115 beats per minute. General examination was otherwise normal.

**Figure 1:**
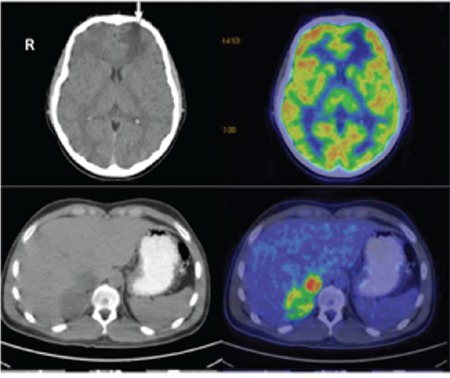
Positron emission tomography imaging showing increased fluorodeoxyglucose uptake in the right adrenal gland, compatible with recurrence, and hypometabolic, hypodense focus in the left frontal lobe

Laboratory tests showed an elevated 24 hour (h) urinary vanillylmandelic acid (VMA) concentration of 115 mg/day (normal value <15 mg/day). An abdominal ultasound revealed solid lesions, 27x35 mm at the right adrenal gland and 37x75 mm at the left adrenal gland. Abdominal magnetic resonance imaging (MRI) showed bilateral adrenal masses compatible with PCC. Bilateral subtotal adrenalectomy, including removal of the masses, was performed and the diagnosis of bilateral PCC was confirmed histologically. The patient remained asymptomatic with no laboratory or radiologic abnormalities for five years of follow-up. At the age of 17, he presented complaining of headache. Cranial MRI demonstrated a lesion of one centimetre diameter, located in the left frontal lobe. Positron emission tomography (PET) revealed a lesion of increased 18 fluorodeoxyglucose uptake in the right adrenal gland, compatible with recurrence, and a hypometabolic, hypodense focus in the left frontal lobe (Figure 1). The cranial mass was excised and hemangioblastoma was diagnosed histologically. Adrenalectomy was performed for the lesion in the right adrenal gland and recurrence of PCC was confirmed. Coexistence of PCC and cranial hemangioblastoma suggested the diagnosis of VHL disease. The previously reported heterozygous missense mutation c.482G>A (p. Arg161Gln) in the *VHL* gene was detected on genetic analysis. 

### Case 2

**Figure 2:**
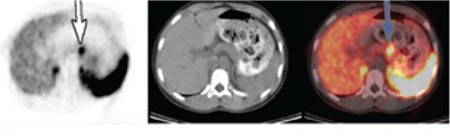
Positron emission tomography - computed tomography imaging showing increased uptake in the right adrenal gland and 11x10 mm nodular lesion in corpus of the pancreas

This 10 year old girl presented with intermittent fever for the past one month. Her birth history was unremarkable. She was the fourth child of nonconsanguineous parents. Her family history was not significant for tumor occurrence. Physical examination revealed a blood pressure level of 160/100 mmHg (95p: 120/79 mmHg) and a heart rate of 110 beats per minute. Laboratory tests showed an elevated 24 h urinary VMA level of 83 mg/day. Abdominal MRI revealed a 44x33 mm, well circumscribed mass with a necrotic core in the left adrenal gland. Subtotal adrenalectomy was performed and histologic examination showed that the tumor was PCC. During two years of follow-up, a 20x19x12 mm mass was detected in the right adrenal gland on abdominal MRI. PET-computed tomography (CT) with ^68^Ga-DOTA‐DPhe^1^, Tyr^3^‐octreotate (^68^Ga- DOTATATE) showed increased uptake in the right adrenal gland and a 11x10 mm nodular lesion in the corpus of the pancreas (Figure 2). The tumoral masses in the adrenal gland and pancreas were removed. Histologic investigation of adrenal and pancreas specimens confirmed the diagnosis of PCC and PNET (World Health Organisation grade 3) respectively. One year later, an 8x7 mm lesion in the pancreas, compatible with recurrence, was observed on abdominal MRI and confirmed with 68 Ga-DOTATATE PET-CT. Splenectomy and subtotal pancreatectomy were performed for removal of the lesion. Histologic examination of the pancreatic lesion reported a neuroendocrine tumor. Bilateral PCC with PNET suggested the diagnosis of VHL disease. Molecular genetic analysis of the *VHL* gene revealed a heterozygous missense mutation c. 386 T>G (p.Leu129Pro) which has been previously described. No additional VHL tumor developed during three years follow up.

Both patients are being followed up according to the recommended pediatric screening protocol for children carrying a VHL mutation (11).

### Genetic Analysis

Molecular DNA was isolated from a 200 µL blood sample using the QIAamp DNA Blood Mini QIAcube Kit with a QIAcube instrument (QIAGEN, Hilden, Germany) according to the manufacturer’s specifications. The full coding sequences, including the 5¢ untranslated region (UTR) and the 3¢ UTR of the *VHL *gene (OMIM*608537), were amplified and sequenced. PCR products were purified using ExoSAP-IT (GE Healthcare, Little Chalfont, UK). The PCR fragments were sequenced by using the BigDye terminator V3.1 Cycle Sequencing ready reaction system (Applied Biosystems, Foster City, CA, USA) according to the manufacturer’s instructions. Sequence analysis was performed on an ABI Prism 3100-Avant DNA sequencer (Applied Biosystems).

## Discussion

PCC is an exceptionally rare neoplasm in children, accounting for 1% of pediatric hypertensive patients (12). Of all PCC cases, approximately 10-20% are reported to occur in the pediatric population (13). Childhood PCC is associated with sustained hypertension, whereas PCC in adults is characterized by hypertensive attacks with the classical triad of palpitation, headache and sweating (14). Episodic tachycardia, sweating and hot flush, the classic symptoms of PCC, accompanied by sustained hypertension, were present in our first patient. However, in the second case, the only symptom was intermittent fever. However, sustained hypertension was detected on physical examination.

PCCs are seen both sporadically and in association with a number of familial cancer syndromes such as VHL disease, multiple endocrine neoplasia type 2, paraganglioma syndromes type 1, 3 and 4, and, rarely, in neurofibromatosis (13). Family history was negative for familial cancer syndromes in both cases. Even in patients with apparently sporadic PCCs, up to 25% will have unsuspected germline mutations. Younger age and multifocal tumors, as in our patients, are significantly associated with the presence of a mutation. Genetic testing may detect patients at risk for other associated tumors (15). The delayed diagnosis of VHL disease was made after the occurrence of cranial hemangioblastoma in the first case and PNET in the second case.

In childhood and adolescence, PCC may be the only initial manifestation of VHL disease with delayed manifestations of the syndrome in the eye, CNS or other organs (16).

VHL disease is classified into four subtypes. type 1 occurs without PCC while type 2A, 2B and 2C all carry the risk of development of PCC. Patients with type 2A have a low risk of renal cell carcinoma (RCC) while Type 2B patients have a high risk of RCC. VHL type 2C confers an increased risk of PCCs without other manifestations of the disease. In type 1 families, deletions in the *VHL* gene are often detected, whereas in type 2 disease, missense mutations are most often encountered. In our cases, the presence of PCC and the missense mutations in *VHL* gene suggested VHL type 2. Mutation found in the first patient, c.482G>A (p. Arg161Gln), is also known to be associated with RCC (17,18). However, to date, our patients have not shown any signs of RCC.

Involvement of the pancreas in VHL disease has been reported in 25% to 70% of cases (19). In most of the cases, pancreatic changes are characterized by benign cysts (20). In VHL disease, neuroendocrine tumors of the pancreas and PCCs are observed in 8-17%, and 10-20% of patients respectively (21). The association of neuroendocrine tumors of the pancreas with PCCs has been reported in 12% of patients with VHL disease (22). Pancreatic tumors rarely occur during childhood (23). The mean age at presentation for neuroendocrine tumours is 35 years (21). In our second case, PNET as a component of VHL was detected at the age of twelve years, two years after diagnosis of PCC. Langrehr et al (24) reported a 12-year-old girl with c.695 G>A mutation in exon 3 of the *VHL* gene resulting in a neuroendocrine tumor of the pancreas and bilateral adrenal PCC. To the best of our knowledge, our patient is the second youngest reported case in the literature, presenting with a combination of PNET and bilateral PCC as components of childhood VHL disease.

To conclude, we have presented two childhood cases of VHL disease with bilateral PCC and an additional tumor, namely PNET and cranial hemangioblastoma diagnosed after two and five years after the initial diagnosis of PCC, respectively. The combination of PCC and PNET in childhood VHL disease is here reported for only the second time in the literature. Meticulous follow-up and early genetic testing in PCC may facilitate diagnosis and serve to prevent morbidity and mortality, as well as improving long term prognosis in VHL disease.
