# Correction

**DOI:** 10.4274/jcrpe.galenos.2020.e001

**Published:** 2020-03-19

Dağdeviren Çakır A, Turan H, Aykut A, Durmaz A, Ercan O, Evliyaoğlu O. Two Childhood Pheochromocytoma Cases due to von Hippel-Lindau Disease, One Associated with Pancreatic Neuroendocrine Tumor: A Very Rare Manifestation. *J Clin Res Pediatr Endocrinol* 2018;10:179-182. Epub 2017 Oct 12

The mistake has been made by author inadvertently. “c.386T>G” word in page 179, Abstract section, line 6 has been corrected as “c.386T>C”.

